# Effect of down-regulation of aquaporins in human corneal endothelial and epithelial cell lines

**Published:** 2010-08-10

**Authors:** Jwalitha Shankardas, Rajkumar V. Patil, Jamboor K. Vishwanatha

**Affiliations:** 1 Department of Biomedical Sciences, Graduate School of Biomedical Sciences, University of North Texas Health Science Center, Fort Worth, TX; 2 Pharmaceutical Research Division, Alcon Laboratories Inc., Fort Worth, TX

## Abstract

**Purpose:**

The purpose of this study was to determine the effects of down-regulation of Aquaporin 1 (*AQP1*) and Aquaporin 5 (*AQP5*) on cell proliferation and migration in human corneal endothelial (HCEC) and human corneal epithelial (CEPI17) cell lines, respectively.

**Methods:**

*AQP1* and *AQP5* were down regulated using siRNA following lipofectamine-mediated transfection in corneal endothelial and epithelial cells, respectively. Down-regulation was confirmed using RT–PCR, indirect immunofluorescence, and immunoblot analysis. Total internal reflection fluorescence (TIRF) microscopy was used to detect cell surface aquaporin expression. Cell proliferation was determined by SRB (sulfrodamine B) assay. Cell migration was determined by in vitro wound healing and migration assay.

**Results:**

In HCEC cells, AQP1 was localized to the cytosol as well as cell membrane and its down-regulation resulted in decreased cell proliferation and migration with a significant decrease in phosphorylated ERK (pERK). In CEPI17 cells AQP5 protein expression was also localized to cytosol as well as cell membrane. AQP5 down-regulation resulted in an increase in proliferation and cell migration with no significant difference in pERK.

**Conclusions:**

AQP1 plays a role in HCEC proliferation and migration via the ERK signaling pathway and therefore may have significant implications in corneal endothelial dysfunction whereas; AQP5 may play an indirect role in human corneal epithelial cell proliferation and migration.

## Introduction

Aquaporins (AQP) are a class of membrane glycoproteins, embedded in the cell membrane, which function mostly as semi-selective pores allowing water to move in response to osmotic and hydrostatic differences [1]. The diverse requirement for the regulation of water homeostasis in different cells and tissues is one of the major reasons for the numerous aquaporins (AQP0-AQP12) that are present [2]. The predominant and well studied function of AQP is the regulation of water transport [3]. Recent studies have implicated other possible functions that include cell migration (angiogenesis, tumor metastasis, wound healing [4], neural function (sensory signaling, seizures) [5] and even differentiation [6]. In the area of vision research, aquaporins have been implicated in certain ocular disorders such as keratoconus [7], bullous keratopathy, and Fuchs' dystrophy [8], and glaucoma [9].

AQP1 and AQP5 both play an important role in the maintenance of corneal hydration Alterations of the amount and/or location of expression of AQP1 and AQP5 have been investigated in a variety of pathological conditions [10-13]. AQP1 is the first molecular water channel that was discovered in red blood cells [14]. In the eye, this protein is expressed in cells from the lens epithelium, corneal endothelium, stroma, trabecular meshwork, and the ciliary non-pigmented epithelium [15]. AQP1 expression levels have been shown to be associated with changes in corneal hydration and maintenance of thickness of the stroma. Changes in corneal thickness can result in impairment of corneal transparency, which is crucial for vision [16]. While it is unclear if down regulation of AQP1 is directly related to corneal endothelial disease, it is suggested that increased AQP1 expression may reduce corneal edema [17]. Maintenance of corneal stromal transparency requires precise regulation of extracellular water content [18]. AQP5 is predominantly expressed in the corneal epithelium and the apical membranes of the lacrimal acini [1]. It is interesting to note that AQP5 is absent in the corneal endothelium where AQP1 is abundantly expressed. AQP5 has been shown to be involved in the maintenance of corneal hydration, which is necessary to maintain corneal transparency [17].

Cell proliferation and migration are two important aspects of wound healing. Overexpression and knock down studies have been extensively used to study the role of AQPs in normal and diseased tissues. AQP1 has also been shown to be involved in cell migration in several cell types such as keratocytes [19], epithelial cells of the kidney [20], and gastric epithelial cells [21]. Recently it was shown that AQP1 contributes to cell migration in endothelial and melanoma cell lines via association with Lin7/β catenin [22]. In addition, AQP5 has been shown to be involved to some extent in cell proliferation and carcinogenesis in colon, gastric, and ovarian cancer tissues [6]. It is however unclear if this is a tissue/cell type specific occurrence. The role of AQP1 and AQP5 in corneal wound healing has not been studied. We examined the role of AQP1 and AQP5 in cell migration and proliferation in the human corneal endothelial cell (HCEC) and SV40 immortalized corneal epithelial (CEPI17) cell lines, respectively. We show here that down regulation of *AQP1* and *AQP5* using target specific siRNA has an impact on cell proliferation and migration and hence, may have implications in wound healing in the cornea.

## Methods

All tissue samples were obtained in compliance with good clinical practice, with informed consent under Institutional Review Board (IRB) regulations, and also in accordance with the tenets of the Declaration of Helsinki.

### Cell culture

#### Primary human corneal epithelial cells

Epithelial sheets were obtained from Eye Bank corneas (after removal of residual sclera and conjunctiva tissue) and the primary cells cultured as previously described [23]. Briefly, donor tissue was incubated in dispase (BD Biosciences, San Jose, CA), diluted with calcium free EpiLife® (Cascade Biologicals, Portland, OR) medium to 12 Units/ml at 4 °C for 48 h. The epithelial sheets were removed from the stromas, dissociated into single cell suspension, and then plated into tissue culture (TC) flasks (75 cm^2^, vented). Flasks were coated with murine collagen type IV (at a concentration of 5 μg/ml) using a cell scraper to evenly coat the entire growth surface. The coated flasks were air-dried under sterile conditions before use (or stored sterile at 4 °C). The cells were cultured in serum free defined media (EpiLife®; Cascade Biologicals) until approximately 80% confluent, and then sub cultured by harvesting with trypsin/EDTA (Gibco BRL, Carlsbad, CA), neutralization of proteolytic activity with trypsin inhibitor (Sigma-Aldrich, St Louis, MO), and plating into freshly collagen IV coated TC flasks (BD Biosciences).

#### Human corneal epithelial cell line

CEPI17 (SV-40 transformed Corneal epithelial cell line) [24] (a kind gift from Dr. Slobodan Dimitrijevich Department of Integrative Physiology, UNTHSC, Fort Worth, TX) were cultured in serum free defined media (EpiLife®; Cascade Biologicals) to 80% confluence. The epithelial cells were sub cultured by harvesting with trypsin/EDTA (Gibco BRL), neutralization of proteolytic activity with trypsin inhibitor (Sigma-Aldrich), and plating into fresh collagen IV coated TC flasks. These cells, also referred to as CEPI-17-CL4, have been considered a suitable corneal epithelial cell model and are currently being widely used [25].

#### Corneal endothelial cell line

HCEC (E6/E7 transformed endothelial cells, a kind gift from Dr. Slobodan Dimitrijevich, Department of Integrative Physiology, UNTHSC, Fort Worth, TX) [26] were cultured in DMEM supplemented with 10% FBS.

### Immunocytochemistry

Approximately 15,000 cells were plated on glass coverslips (12 cm^2^, Thermofisher; Fisher Scientific, Pittsburgh, PA) and cultured in their respective media. When the cultures had stabilized, the coverslips were rinsed in PBS, and fixed/permeabilized in methanol: acetone (1:1, 10 min at −20 °C). After re-hydration in phosphate buffered saline (PBS, 0.256 g/l NaH_2_PO_4_ H_2_O, 1.19 g/l Na_2_HPO_4_, 8.76 g/l NaCl, pH 7.4; for 30 min), and distilled water washes (3×) the cells were blocked (overnight at 4 °C) in PBS + 1% BSA (BSA). The cells were then rinsed with PBS and distilled water (3×), and incubated at 4 °C, overnight, with primary (1°) antibody diluted in PBS. After rinsing in PBS containing Tween-20 (0.1%; 3×10 min), cells were incubated with secondary (2°) antibody at room temperature (RT, 1.5 h) and rinsed in PBS + Tween-20 (0.1%, 3×10 min). The specimens were rinsed in PBS (3×10 min), distilled water (30 min), stained with 4’, 6-diamino-2-phenylindole (DAPI) and mounted on glass slides (FluorSave; Calbiochem, La Jolla, CA). Cells on coverslips stained with secondary antibody alone were used as negative controls, which showed virtually no fluorescence.

### Antibodies

Anti-AQP5, anti-AQP1 and anti-GAPDH primary antibodies were purchased from Santa Cruz Biotechnology (Santa Cruz, CA). Anti-ERK, anti- phosphorylated ERK antibodies were purchased from BD biosciences. Alexa Fluor 594 nm goat anti-mouse, Alexa Fluor 594 goat-anti-rabbit and Alexa Fluor 594 nm donkey anti-goat (Molecular Probes/Invitrogen, San Diego, CA) secondary antibodies were used at dilutions of 1:1,000. Negative controls in all experiments were specimens labeled with 2° antibody only and DAPI to show nuclei; these showed virtually no background fluorescence.

### Image acquisition

Mounted specimens were examined on Olympus AX70 fluorescent microscope (Olympus, Center Valley, PA) using SPOT Twain software (Microsoft, Issaquah, WA).

### Transfection

Cells were cultured and transfected using Lipofectamine 2000 (Molecular Probes/Invitrogen) when they were 70% confluent. Transfection was performed as per manufacturer’s instructions. CEPI17 and HCEC cells were transfected with 100 nM concentration of On-TARGET plus SMART pool AQP5 (Dharmacon, Pittsburgh, PA) and On-TARGET plus SMART pool AQP1 (Dharmacon), respectively. Transfection of cells with 100 nM Non Targeting siRNA#3 (siGENOME® Control siRNA; Dharmacon) served as a transfection control. Transfected cells were then used for further analysis.

### RNA isolation and RT–PCR

RNA was isolated using Trizol® reagent according to manufacturer’s recommendations for cells cultured as monolayer (Invitrogen). Trizol (1 ml/cm^2^ of cell monolayer) was added to the washed cell monolayer and the cells detached from the flask using a cell scraper. The cells mixture was transferred to centrifuge tubes and incubated at 30 °C for 5 min and then scraped. To this 0.2 ml of chloroform was added and the closed tube shaken vigorously by hand for 15 s, opened and incubated at 30 °C for 2–3 min. The sample was then centrifuged at no more than 12,000× g (2–8 °C) for 15 min. The top aqueous layer was carefully removed and transferred to a fresh centrifuge tube and the RNA precipitated with isopropanol (0.5 ml of isopropanol/ml of Trizol reagent used). The precipitated RNA was washed twice with ethanol and finally dissolved in DEPC treated water. Primers used were as follows: AQP5 (forward: 5′-AAG GCC GTG TTC GCA GAG TT-3′, reverse: 5′-TGG TCA GCT CCA TGG TCT TC-3′, annealing temperature: 55 °C); AQP1 (forward: 5′-CTT ACC TCC AGG ACC CTT CC-3′,  reverse: 5′-TAG CTC ATC CAC ACG TGC TC-3′, annealing temperature: 60 °C). RT–PCR was performed using Superscript® One step RT–PCR system (Invitrogen). Agarose gel (1.2%) was prepared by heating agarose in TAE buffer. After cooling, ethidium bromide (6 μl in 100 ml of solution) was added to the mixture. The total sample obtained from RT–PCR was loaded on to the gel with 5 μl of bromophenol blue dye and imaging was performed using UVP imaging device.

### Western blot analysis

Cultured epithelial and endothelial cells were treated with lysis buffer (2.5 ml 1 M Tris buffer [pH=7.0], 1 g SDS, and 2.5 g sucrose in 50 ml distilled water) for 5 min at RT as previously described [26]. Genomic DNA was sheared by several passes through a 22-gauge needle, and samples stored at –20 °C until needed. BCA (bicinchoninic acid) protein assays (Pierce, Rockford, IL) of lysates were performed to determine the protein concentration (and ensure equal loading of lanes). SDS PAGE was performed at RT, and 20 μg protein/lane was loaded with 12% Tris-Glycine, at 150 V in Tris/glycine as the running buffer. Protein bands were transferred onto nitrocellulose membranes (VWR International, Irving, TX) by electro-blotting overnight (4 °C) at 10 V in Tris/glycine buffer with 20% methanol and transfer was confirmed with Ponceau Red (Sigma-Aldrich) staining of the membranes. After de-staining in distilled water, membranes were incubated in blocking buffer (5% powdered milk and 1% BSA in PBS) for 1 h (RT). Membranes were then incubated with 1° antibody for 30 min (RT), then overnight (4 °C) and finally the following morning for 30 min (RT). After rinsing in PBS containing 0.1% Tween-20 (3×10 min), the membranes were incubated with 2° antibody for 1 h (RT), rinsed in PBS with 0.1% Tween-20 (3×10 min), and developed (ECL Chemiluminescence; Amersham Biosciences, Little Chalfont, UK). Densitometric analysis was performed on the western blot images using Image J (nih.gov) to quantitate the amount of mRNA and protein levels following transfection with target specific siRNA at different time points.

### TIRF microscopy

Primary human corneal epithelial and CEPI17 cells were grown on coverslips and mounted on 24×60 cover glass (Falcon#1; Fisher Scientic, Pittsburgh, PA). Unpermeabilized cells were immunostained with combinations of primary antibodies and Alexa-fluor secondary antibodies (1:1,000; Molecular probes). We also used a cytosolic protein (tubulin) to eliminate cytosolic epi-fluorescence. The cells were examined under the Olympus IX71 microscope (Olympus, Center Valley, PA) equipped with a commercial TIRF attachment (60×) at the Center for Commercialization of Fluorescence Technologies (CCFT), UNTHSC.

### In vitro wound healing assay

Cells were seeded at high density in 6 well plates 72 h after transfection with siRNA. Two (or more) parallel scratch wounds of approximately 400 μm width were made perpendicular to the marker lines with a yellow P200 pipette tip (Fisher). This procedure makes it possible to image the entire width of the wound using a 4× objective. The wounds are observed using phase contrast microscopy on an inverted microscope. Images were taken at regular intervals over the course of 0–36 h of both areas flanking the intersections of the wound and the marker lines (5 images per experimental condition). The width of the wound was controlled so that it is as consistent as possible, since narrow wounds tend to close faster than wider wounds. If more than two scratches were made, wounds with similar widths were chosen for analysis. Images were analyzed by digitally drawing lines (using Image J software) averaging the position of the migrating cells at the wound edges. The cell migration distance was determined by measuring the area of the wound under different experimental conditions and compared to the control.

### In vitro invasion assay

HCEC cells both control and those transfected with AQP1 siRNA were treated with Cell Tracker Green® for 2 h (Invitrogen, Carlsbad, CA). The cells were then seeded onto Matrigel endothelial cell migration chambers (BD Biosciences, San Jose, CA) 72 h after transfection. These chambers were hydrated for at least 2 h in the tissue culture incubator with 500 μl serum free DMEM in the bottom of the well and 500 μl in the top of the chamber. After hydration of the Matrigel, the DMEM in the bottom of the well was replaced with DMEM containing 10% FBS. HCEC cells (500,000) were plated in 500 μl of DMEM supplemented with 10% FBS in the top of the chamber. The invasion assay was performed for 24 h in the tissue culture incubator. The cells were fixed by replacing the culture medium in the bottom and top of the chamber with 4% formaldehyde dissolved in PBS. The cells that migrated were counted using an inverted microscope equipped with either a 4× or a 10× objective and plotted as the percentage of invading cells of the total number of plated cells.

### Sulfrodamine B (SRB) cell proliferation assay

The SRB proliferation assay was performed as described earlier [27]. Briefly, control and transfected cells (CEPI 17 and HCEC) were seeded in a 96 well plate at a cell density of 5,000 cells/well 24, 48, and 96 h after transfection. On days 2, 3, and 5, the media was removed and cells were fixed with 10% TCA for 10 min at 4 °C. The cells were washed 4 times with distilled water, and then stained 10 min with SRB dissolved in 1.0% acetic acid. The plates were washed 4 times with 1.0% acetic acid and allowed to air dry. At this point they were stable and left overnight or longer at room temperature. Prior to reading the plates, 200 µl of 10 mM Tris base (unbuffered) was added per well to solublize the dye. The plates were mixed for 5 min on a Lab Line Instruments Titer Plate Shaker (VWR International, Irving, TX) and read at 564nm on a Molecular Devices SpectraMax 340 (Molecular Devices, Sunnyvale, CA), using Soft Pro Max acquisition software (Molecular Devices). Absorbance data were exported into a Microsoft Excel spreadsheet (Microsoft) for further analysis. Cell densities were obtained by correcting absorbances for media controls and used for statistical analysis.

### Statistical analysis

Statistical analysis was performed using paired Student’s *t*-test and a p-value <0.05 was taken as statistically significant for all the experiments.

## Results

### Sub-cellular localization of AQP1 in HCEC cells

**Figure 1:**
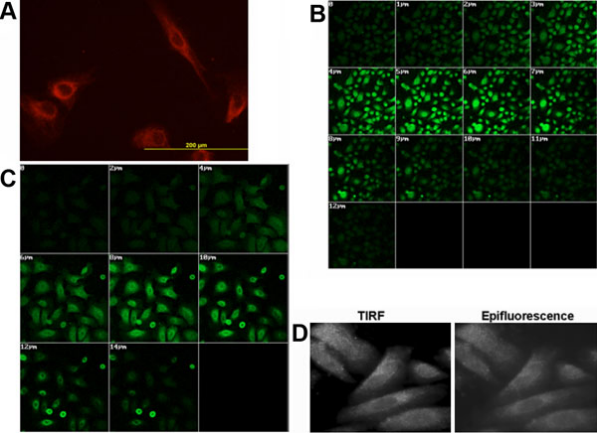
Immunocytochemical localization analysis of AQP expression in corneal cell lines. The panels show (**A**) AQP1 expression (red) in HCEC cells, (**B**) Z-sectioning analysis showing AQP5 expression (green) in CEPI17 cells, and (**C**) Z-sectioning analysis showing AQP5 expression (green) in Primary WT CEPI cells. TIRF microscopic analysis showing the membrane association of AQP5 in CEPI17 cells is seen in panel **D**.

The localization of AQP1 was investigated by immunocytochemistry using mouse AQP1 monoclonal antibody in HCEC cells. HCEC cells stained with only fluorescent secondary antibody served as negative controls. As shown in Figure 1A, AQP1 was detected in the cytosol as well as membrane-associated regions of the HCEC cells as observed by fluorescence microscopic analysis. Negative controls showed virtually no fluorescence (data not shown).

### Sub-cellular localization of AQP5 in CEPI17 cells

The cellular and sub cellular localization of the AQP5 protein in CEPI17 cells was investigated by immunocytochemistry, using rabbit polyclonal anti-AQP5 antibody. As seen in Figure 1B, AQP5 is localized to both cytosol as well cell membrane regions in CEPI17 cells as shown by Z-sectioning analysis (14 sections at 2 μm/section from the top of the cell to the bottom) performed using confocal microscopy (Figure 1B). Sections at 2 μm, 3 μm, 7 μm, and 8 μm represent the membrane associated AQP5 and sections 4 μm-7 μm represent the cytosolic region of the cells. Primary corneal epithelial cells (Figure 1C) were used as positive controls to show similar localization of AQP5 in the CEPI17 cells. No fluorescence was detected in the negative controls (data not shown). Furthermore, as shown in Figure 1D, we also performed total internal reflection (TIRF) microscopy followed by immunocytochemistry to study the cell membrane distribution of AQP5. AQP5 is abundantly expressed at distinct spots on the cell membrane in comparison to the epifluorescence image where the fluorescence appears to be diffuse and cytosolic. The cells were also immunostained with a cytosolic protein, tubulin followed by TIRF microscopy to serve as a negative control (data not shown).

### Effective down-regulation of *AQP1* using siRNA in HCEC cells

**Figure 2:**
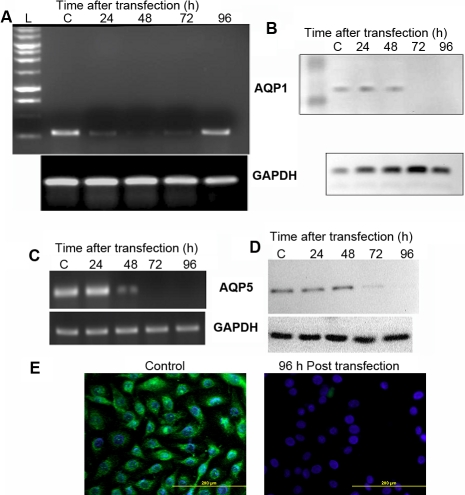
Effective silencing of *AQP1* and *AQP5* using siRNA. Down-regulation of *AQP1* expression in HCEC cells using siRNA as determined by (**A**) RT–PCR and (**B**) western blot analysis. Down-regulation of *AQP5* expression in CEPI17 cells using siRNA as determined by (**C**) RT–PCR, (**D**) western blot analysis. Panel **E** shows down-regulation of AQP5 (green) in CEPI17 cells, 96 h post trasnfection with *AQP5* siRNA. AQP5 down regulation was determined by Immunocytochemical analysis (DAPI was used to stain the nucleus).

HCEC cells were transfected with *AQP1* siRNA and scrambled control siRNA using lipofectamine. Significant down-regulation (98%) in the *AQP1* mRNA level was observed in 48 h (lane 3, Figure 2A) following transfection with *AQP1* siRNA as determined by RT–PCR analysis. The mRNA levels, however, increased at the 96 h time point and hence all the downstream experiments were conducted 72 to 96 h after transfection. Significant down-regulation (97%) in AQP1 protein synthesis was also observed 72 h (lane 5, Figure 2B) after transfection with *AQP1* siRNA. This down-regulation was determined by western blot analysis using AQP1 antibody and the experiments were repeated three times (n=3).

### Effective down-regulation of *AQP5* using siRNA in CEPI17 cells

CEPI17 cells were transfected with *AQP5* siRNA and scrambled control siRNA. There was significant down-regulation (96%) in the *AQP5* mRNA levels 48 h post transfection (lane 3, Figure 2C) in cells transfected with *AQP5* siRNA as determined by RT–PCR analysis. Significant down regulation (90%) in AQP5 protein synthesis was also observed after 72 h (lane 4, Figure 2D) following transfection with *AQP5* siRNA as determined by western blot analysis repeated three times. This result was also confirmed by indirect immunofluorescence using polyclonal anti-AQP5 antibody 96 h following transfection with *AQP5* siRNA. As shown in Figure 2E, there is minimal expression of AQP5 (green) in the cells that were transfected with *AQP5* siRNA, whereas the expression of AQP5 is unaltered in the control cells.

### Effect of *AQP1* down-regulation on HCEC migration and proliferation

**Figure 3:**
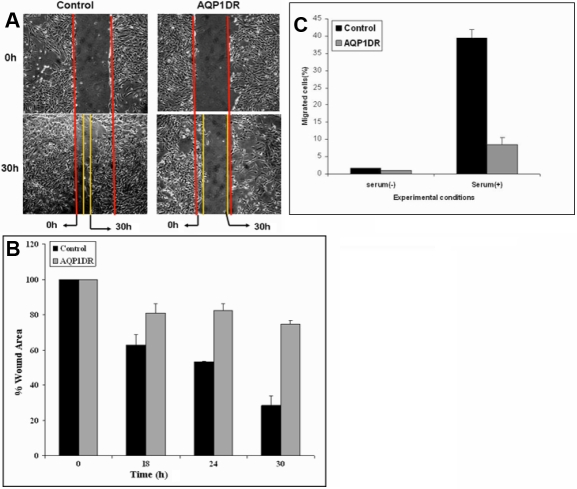
Effect of siRNA mediated *AQP1* down-regulation (AQP1DR) on cell migration in HCEC cells. Panels **A** and B show that AQP1DR decreased cell migration as determined by the scratch wound healing assay in control and AQP1DR cells. A decrease in cell migration was significant at 24 h and 30 h p<0.05 and CI=95% as determined by the paired T-Test (n=3). **C**: Decreased cell migration was observed in HCEC cells following AQP1DR as compared to control cells, as determined by the in vitro endothelial cell migration assay.

**Figure 4:**
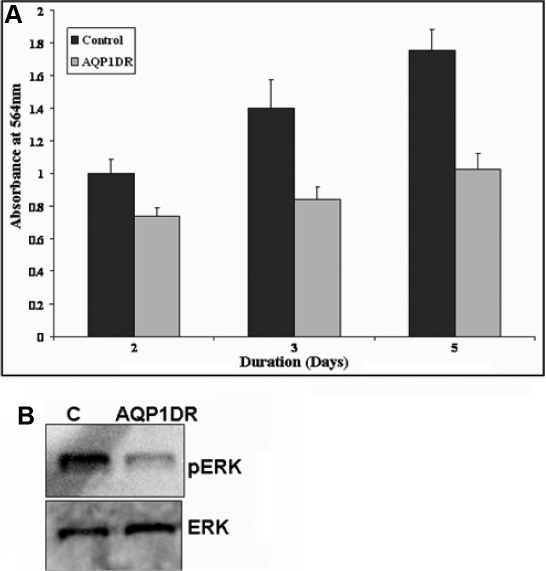
Effect of siRNA mediated *AQP1* down-regulation (AQP1DR) on cell proliferation in HCEC cells. **A**: SRB (Sulforhodamine B) assay was used to determine the effect *AQP1* down regulation (AQP1DR) on HCEC proliferation. AQP1DR decreased HCEC cell proliferation (significant at 3 and 5 days p<0.05, 95% CI as determined by the paired T-Test, [n=3]). **B**: Decrease in phosphorylated ERK 96 h post AQP1DR is shown by western blot analysis in HCEC cells.

*AQP1* siRNA was used to study the effect of down regulation of *AQP1* on migration of HCEC cells. HCEC cell line was transfected with *AQP1* siRNA and wound-healing assay was performed to study the effect of *AQP1* siRNA on the migration of HCEC cells. As seen in Figure 3A, *AQP1* down regulation resulted in decrease in migration of HCEC cells as determined by in vitro wound healing assay. However, when the control siRNA was used to transfect the HCEC cells it had no effect on cell migration (Figure 3A). The area of the wound over time was measured using image J software as described earlier and statistical analysis was performed. There was significant decrease in cell migration 30 h after the scratch was created (Figure 3B). The scratch assay was also performed in the presence of 0.04% Mitomycin C, an inhibitor of cell proliferation, and similar results were obtained (data not shown). The effect of *AQP1* down regulation on cell migration was also studied using the in vitro matrigel cell migration assay (Figure 3C). As shown in Figure 3C, a significant decrease in cell migration was observed in HCEC cells transfected with *AQP1* siRNA. HCEC cells transfected with scrambled siRNA were used as controls. To study the effect of *AQP1* down regulation on cell proliferation, SRB assay was performed as described earlier. Significant decrease in cell proliferation was also observed on *AQP1* down-regulation as determined by SRB proliferation assay (Figure 4A). There was significant decrease in cell proliferation after 3 and 5 days following transfection with *AQP1* siRNA. ERK phosphorylation was monitored as a marker for cell proliferation. There was also a significant decrease in levels of phosphorylated ERK protein in *AQP1* down-regulated cells when compared to the controls 96 h post transfection (Figure 4B). There was no change in the levels of phosphorylated ERK at other time points (data not shown). This was determined by western blot analysis using phosphorylated ERK antibody. Expression levels of total ERK was used as a loading control.

### Effect of *AQP5* down-regulation on CEPI17 migration, proliferation and ERK phosphorylation

**Figure 5:**
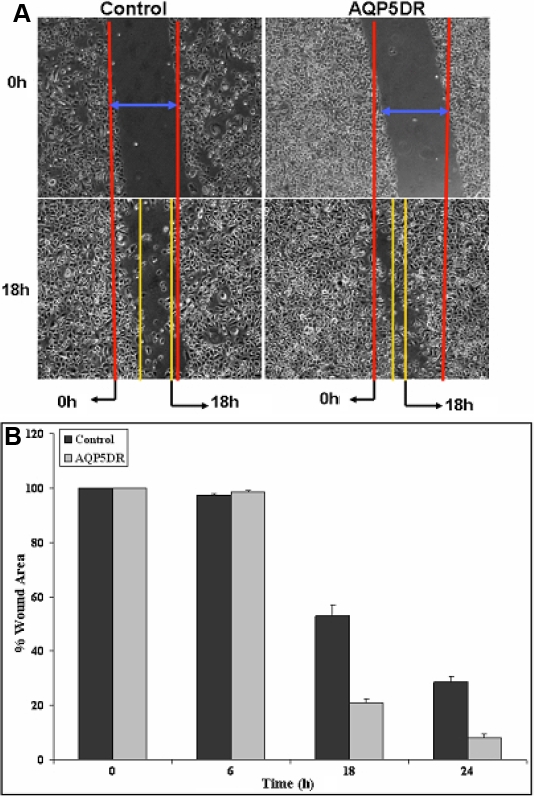
Effect of siRNA mediated *AQP5* down-regulation (AQP5DR) on cell migration in CEPI17 cells. Panels **A** and **B** show that AQP5DR decreased cell migration as determined by the scratch wound healing assay in control and AQP5DR cells. The decrease in cell migration was significant at 24 h and 30 h p<0.05 and CI=95% as determined by the paired T-Test, [n=3]).

**Figure 6:**
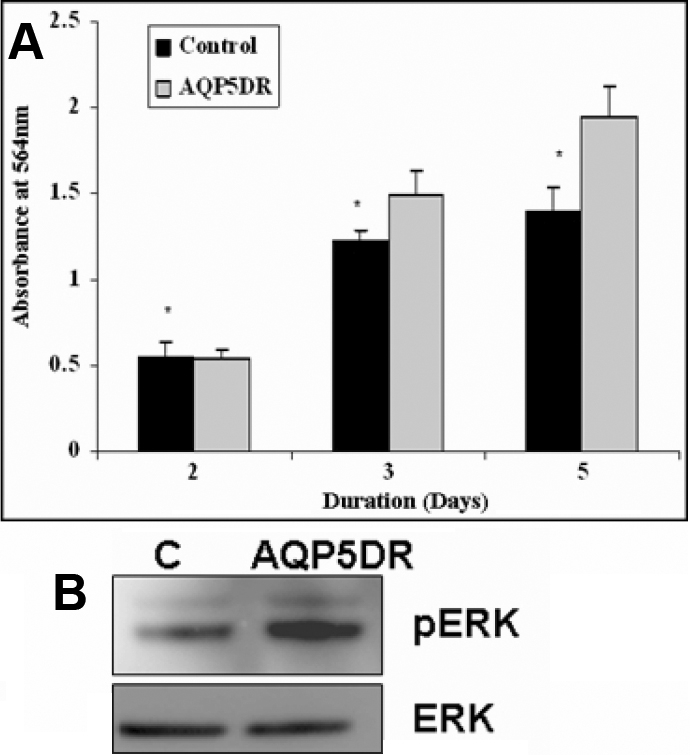
Effect of siRNA mediated *AQP5* down-regulation (AQP5DR) on cell proliferation in CEPI17 cells. **A:** SRB assay shows that AQP5DR increased cell proliferation in CEPI17 cells (significant at 3 and 5 days p<0.05, 95% CI as determined by the paired T-Test, [n=3]). There was no significant difference in phosphorylated ERK 96 h post transfection in CEPI17 cells when compared to the controls as shown by western blot analysis (**B**).

*AQP5* siRNA was used to study the effect of down regulation of *AQP5* on migration of CEPI17 cells. CEPI17 cells were transfected with *AQP5* siRNA and wound-healing assay was performed to study the effect of *AQP5* siRNA on the migration of CEPI17 cells as described earlier. As seen in Figure 5A, *AQP5* down regulation resulted in significant increase in migration of CEPI17 cells as determined by the in vitro wound-healing assay. However, when control siRNA was used to transfect the CEPI17 cells it had no effect on cell migration (Figure 5A). The area of the wound over time was measured using image J software and statistical analysis was performed. There was significant increase in cell migration 18 h after the scratch was created (Figure 5A,B). The scratch assay was also performed in the presence of 0.04% Mitomycin C, an inhibitor of cell proliferation, and similar results were obtained (data not shown). To study the effect of *AQP5* down regulation on cell proliferation, SRB assay was performed as described earlier. Significant increase in cell proliferation was also observed on *AQP5* down-regulation as determined by SRB proliferation assay (Figure 6A). This was significant after 3 and 5 days following transfection with *AQP5* siRNA. ERK phosphorylation was monitored over time as a marker for cell proliferation. There was, however, no significant change in levels of phosphorylated ERK protein in *AQP5* down-regulated cells when compared to the controls at different time points (96 h time point shown in Figure 6B, repeated three times). This was determined by western blot analysis using phosphorylated ERK antibody. Expression levels of total ERK was used as a loading control.

## Discussion

We used HCEC and CEPI17 cell lines to investigate the localization of AQP1 and AQP5. Primary corneal endothelial and epithelial cells are extremely hard to culture and have a very limited proliferative life span in vitro, which depends on the age of the donor [28]. This was the main reason for opting to use the CEPI17 cell line, which is a commonly used corneal epithelial cell model [24]. Further, availability of tissue from younger donors is very limited and hence the HCEC and CEPI17 cell lines were used in our expression and down regulation studies. Primary corneal epithelial cells had similar AQP5 expression as the CEPI17 cells; this helped ascertain that the CEPI17 was a suitable model for the described studies. Primary endothelial cells are extremely hard to culture and hence, they were not used in our experiments due to their lack of availability. Our studies on localization of AQPs have shown that both AQP1 and AQP5 are localized both to the cytosol and cell membrane. To localize the presence of AQP5 in CEPI17 cells, TIRF microscopy was used. TIRF microscopy or evanescent wave microscopy is a specialized type of confocal microscopy which allows the excitation of fluorophores within a closely restricted zone (70 nm) of the plasma membrane increasing the signal to noise ratio of the plasma membrane to cytosolic fluorescence. This type of microscopy is commonly used to study plasma membrane associated events [29]. Previously, localization of AQP1 and AQP5 has been shown to be membrane associated in vivo [30]. Our observation of localization of AQP1 and AQP5 in both cytosol as well as membrane could be tissue/cell specific or a purely in vitro phenomenon. Localization of the AQPs has been shown to affect secretory function of tissues such as salivary glands, lacrimal glands, and the pancreas in Sjorgen's syndrome [11]. Studies have shown changes in protein localization in normal versus diseased tissues from donors with Sjorgen's syndrome [31], there are however some contradicting studies in this area [32]. Hence, localization of these proteins may have considerable impact on function even in ocular tissues.

Our results demonstrate that there is a significant decrease in cell migration and proliferation in HCEC cells following *AQP1* down regulation. Using siRNA we could significantly down regulate the *AQP1* mRNA and protein levels. We have performed a detailed time course study to determine the time point where there is maximum down-regulation at the protein level for our downstream experiments. There is however a reoccurrence of *AQP1* mRNA at 96 h following lipofectamine mediated transfection with *AQP1* siRNA. This could be due to two possible reasons: (1) The silencing was only transient and after 96 h the siRNA mediated silencing was abrogated beyond that time point, (2) AQP1 is an osmoinducible water channel and the *AQP1* gene has a hyper tonicity-responsive element [33]. It is therefore possible that there is a resurgence of AQP1 at 96 h in response to changes in cellular tonicity, but this needs further investigation. However, since the experiments we have described are performed at the time point when there is significant down regulation of *AQP1*, the reoccurrence of the mRNA after 96 h following transfection will not affect our data.

Down regulation of *AQP1* has been shown to be involved in decreased cell migration in several cell types including human melanoma cell lines, human microvascular endothelial cells, kidney epithelial cells, and gastric epithelial cells. Overexpression of *AQP1* has been reported in proliferating tumor vessels suggesting its involvement in tumor angiogenesis [34]. However, corneal endothelial cells are different from vascular endothelial cells as they are derived from the neural crest and are in fact leaky epithelial cells. Also, the proliferative capacity of normal human corneal endothelial cells depends on the age of the donor and it decreases with the age of the donor [28]. Also, corneal endothelial cells, as compared to vascular endothelial cells, have a different function, which is to act as a water pump, and therefore these cells do not form blood vessels. The results however show a similar response to *AQP1* down-regulation as the vascular endothelial cells that have been previously reported [22].

Our data also show a significant increase in cell proliferation and migration upon *AQP5* down-regulation in a corneal epithelial cell line (CEPI17). Previous studies have shown an upregulation of *AQP5* in ovarian tumors [35], colorectal carcinomas [10], and in human small cell lung cancer [36]. These studies have also shown the involvement of the ERK signaling cascades in response to changes in *AQP5* expression levels [37]. However, we did not observe significant increase in phosphorylated ERK, which implies the involvement of an alternate proliferation pathway that needs to be explored. This could be a tissue specific event considering the fact that the cornea is an immune privileged site and corneal cancers are rare. It has been reported that AQP3 plays a major role in corneal re-epithelialization and AQP3-deficient mice have defects in corneal epithelial cell migration and proliferation [38]. It is not known and further studies are required to determine whether down-regulation of *AQP5* could cause an increased expression of *AQP3*, which could lead to increased cell proliferation and migration.

In both cell types (CEPI17 and HCEC) we did not observe any significant changes in cell size and cell morphology following *AQP* down regulation as reported previously in primary cultures of astrocytes, where there was significant decrease in cell size following *AQP4* down regulation [39,40]. However, this phenomenon was observed only in vitro culture following transfection with *AQP4* siRNA, however, the astrocytes from *AQP4* knock out models were similar in size and morphology to normal controls [39,41].

The numerous functions of AQPs in tissue homeostasis and disease are currently being explored for clinical benefits. Currently, AQP targeting drugs are also being considered to be of potential benefit as diuretics and in treatment of conditions such as epilepsy, brain edema, glaucoma, wound healing, obesity and cancer. Our data provides evidence that AQP1 and AQP5 could play an important role in corneal wound healing. Down regulation of *AQP1* in HCEC decreases ERK dependent proliferation and migration suggesting *AQP1* stimulators may have significant implications in corneal wound healing and neovascularization. However, down regulation of *AQP5* increases the proliferation and migration of CEPI17 cells suggesting *AQP5* inhibitors may have significant implications in corneal wound healing.
